# Early differences in auditory processing relate to Autism Spectrum Disorder traits in infants with Neurofibromatosis Type I

**DOI:** 10.1186/s11689-021-09364-3

**Published:** 2021-05-28

**Authors:** Jannath Begum-Ali, Anna Kolesnik-Taylor, Isabel Quiroz, Luke Mason, Shruti Garg, Jonathan Green, Mark H. Johnson, Emily J. H. Jones, the STAARS and EDEN Teams, Rebecca Holman, Sarah Kalwarowsky, Laura Pirazzoli, Chloë Taylor, Grace Vassallo, Emma Burkitt-Wright, Judith Eelloo, D. Gareth Evans, Siobhan West, Eileen Hupton, Lauren Lewis, Louise Robinson, Angus Dobbie, Ruth Drimer, Saghira Malik Sharif, Helen Bethell, Rachel Jones, Susan Musson, Catherine Prem, Miranda Splitt, Karen Horridge, Diana Baralle, Carolyn Redman, Helen Tomkins

**Affiliations:** 1 Centre for Brain and Cognitive Development, Birkbeck, University of London, Henry Wellcome Building, Malet Street, London, WC1E 7HX UK; 2 Medical Research Council Cognition and Brain Sciences Unit, University of Cambridge, Cambridge, UK; 3 Division of Neuroscience and Experimental Psychology, University of Manchester, Manchester, UK; 4 Department of Psychology, University of Cambridge, Cambridge, UK

**Keywords:** Neurofibromatosis type 1, Auditory processing, Habituation, Change detection, EEG, Autism spectrum disorder

## Abstract

**Background:**

Sensory modulation difficulties are common in children with conditions such as Autism Spectrum Disorder (ASD) and could contribute to other social and non-social symptoms. Positing a causal role for sensory processing differences requires observing atypical sensory reactivity prior to the emergence of other symptoms, which can be achieved through prospective studies.

**Methods:**

In this longitudinal study, we examined auditory repetition suppression and change detection at 5 and 10 months in infants with and without Neurofibromatosis Type 1 (NF1), a condition associated with higher likelihood of developing ASD.

**Results:**

In typically developing infants, suppression to vowel repetition and enhanced responses to vowel/pitch change decreased with age over posterior regions, becoming more frontally specific; age-related change was diminished in the NF1 group. Whilst both groups detected changes in vowel and pitch, the NF1 group were largely slower to show a differentiated neural response. Auditory responses did not relate to later language, but were related to later ASD traits.

**Conclusions:**

These findings represent the first demonstration of atypical brain responses to sounds in infants with NF1 and suggest they may relate to the likelihood of later ASD.

**Supplementary Information:**

The online version contains supplementary material available at 10.1186/s11689-021-09364-3.

## Background

Autism Spectrum Disorder (ASD) is characterised by difficulties in social communication and restrictive and repetitive behaviours (DSM-5 [2];). A range of underlying genetic and environmental aetiologies have been identified, but the pathways that link these distal causal factors to the later emergence of diagnostic symptoms remain unclear [16]. Recently, sensory symptoms have been recognised as a core part of the diagnostic profile [2]. Indeed, up to 90% of individuals with ASD report difficulties in sensory processing [65] that include both hyper and hyposensitivity to auditory, tactile, and visual stimulation [90]. Since sensory systems mature very early in postnatal development, it is possible that early sensory atypicalities have a cascading effect on developmental trajectories and contribute to later emerging behavioural symptoms [55, 68]. However, there remains little direct investigation of this possibility.

Identifying causal pathways to symptom emergence requires prospective longitudinal studies of infants with an elevated likelihood of developing ASD. One common approach has been to study infants with an older sibling with ASD [56, 84, 91]. Such studies have identified some evidence of early sensory atypicalities in infants with later ASD, including faster identification of visual differences [18, 40], slower latency of pupillary responses to luminance changes [80], increased behavioural responses to perceptual change [19], and elevated cortical reactivity to repeated sounds [60]. This range of work suggests that early disruptions in sensory processing may be detectable in infants with later ASD prior to the onset of other behavioural symptoms, consistent with a causal model. Observations from familial designs, however, are limited to the 10–20% of children whose ASD is associated with the accumulation of multiple common genetic variants of small effect [64, 94]. It is not currently clear whether similar effects are present in the 5–11% of autistic children who present with a monogenic or more penetrant cause of ASD [108], or indeed in idiopathic cases of ASD where there is a non-familial route to the disorder.

A complementary approach to familial designs is thus to study infants who have an elevated likelihood of developing ASD due to the presence of a monogenic disorder that can be identified in infancy. A strong candidate monogenic condition associated with ASD is Neurofibromatosis Type 1 (NF1), an autosomal dominant neurocutaneous disorder with a birth incidence of 1:2700 [28]. Fifty percent of cases of NF1 are inherited, while the rest arise de novo due to spontaneous loss-of-function mutation of the NF1 gene located on chromosome 17q11.2 [20]. The NF1 gene encodes for neurofibromin, a large 2818-amino acid negative RAS GTPase-regulating protein [97, 98]. Although the physical phenotype of NF1 may include neurofibromas, café-au-lait macules, Lisch nodules and abnormalities within the skeleton and the central nervous system [54], the main challenges reported by parents and children with NF1 in clinical settings are cognitive, social and behavioural difficulties [44, 75]. Indeed, up to 25% of children with NF1 may meet criteria for ASD, up to 45% may experience broader autism symptomatology [34, 36, 87, 109] and up to 50% receive a diagnosis of ADHD [53, 61].

NF1 is a suitable monogenic condition for studying early developmental pathways to ASD for several key reasons. First, the phenotypic profile of ASD in NF1 is broadly similar to idiopathic ASD [37], with a similar male bias in prevalence of ASD [35], making insights from NF1 more likely to be generalisable to the understanding of ASD as a whole. Second, NF1 is typically identified early in development through either cord blood testing in familial cases or through its cutaneous manifestations in both inherited and de novo cases (particularly café-au-lait spots). This makes prospective studies from early infancy feasible. Third, NF1 is not associated with profound developmental delays, but rather with a more subtle shift in IQ [53]. Due to this, comparisons with typically developing infants are less confounded by selection bias and developmental challenges within the NF1 group [3]. Finally, there are good animal models of NF1 that may facilitate the subsequent investigation of neural mechanisms underlying particular phenotypes in human infants [22, 43, 103]. Such investigations may highlight new paths in animal-to-human translation.

One promising domain of investigation in infants with NF1 is low-level auditory processing. Important to pathways to translational research, auditory paradigms can be meaningfully reproduced across the lifespan and in animal models [8, 99] where the importance of auditory processing is high across species (unlike visual processing, which is far more important in primates than in rodents). In particular, the suppression of neural responses following repetition and an increase in response when change is detected are suitable for studying auditory brain development across the lifespan [76, 86]. Failure to attenuate responses to repetition or to respond selectively to a change in auditory input could compromise language development [6, 7] and may also relate to broader aspects of cognitive inflexibility, which has been noted in several neurodevelopmental disorders including ASD, Prader-Willi, Rett Syndrome and Fragile X [2, 66, 70, 81]. Further, failure in these basic learning mechanisms may indicate alterations in neural organisation, consistent with observations of atypical neural connectivity in developmental disorders as well as differences in the co-ordination of excitation and inhibition [24, 27, 67, 92].

Previous work has indeed identified alterations in auditory responses in children with NF1. A study of 22 children and adults with NF1 found that while peripheral acoustic hearing was within the normal range, differences emerged in temporal auditory processing during standardised tests, including phonological processing and temporal resolution [4]. Difficulties in auditory processing were further associated with degree of language impairment and communication disorders in the sample. Additionally, Chaix et al. [14] found that children with NF1 (*n* = 75) scored lower on a phoneme deletion task, indicating impaired phonological processing. However, there has been no work on early auditory development in infants with NF1, and no efforts to examine the relationship between auditory processing and the presence of ASD within the sample.

One common method that has been used to measure responses to auditory repetition and change in the developing brain is electroencephalography, or EEG. EEG is suitable for infants and children of all ages because it is relatively non-invasive and does not require verbal or behavioural responses. The high temporal resolution of EEG also allows it to accurately capture the time-course of neural correlates of auditory processing. EEG studies have shown that neural responses to auditory stimuli decrease with repetition and that the magnitude of this effect increases with age [14]. A related phenomenon is sensory gating, in which a pair of stimuli are presented in quick succession after a period of silence and the second stimulus elicits a smaller neural response than the first [45]. Importantly, individual differences in responses to repetition have been related to future cognitive abilities of the infant [69], as well as neurodevelopmental conditions such as ASD [46, 60, 67, 100]. Atypical neural responses to repetition in ASD have also been linked to severity of behavioural symptoms [83].

Another fundamental feature of low-level auditory processing is the ability to detect when changes occur in a sequence of repeated tones. The mismatch negativity is a frontocentral negative deflection in an event-related waveform, elicited by subtracting responses to an infrequent “deviant” from a repeatedly presented “standard” tone [5, 17, 48, 62, 63, 96]. Two- to 4-month-old infants show age-related differences in responses to deviant versus standard tones, with a slow positive wave at 2 months that becomes an adult-like negativity in 3–4-month-olds [48]. When embedded within a train of repeating stimuli, infants have shown sensitivity to both pitch [32, 48, 110] and frequency [9, 82] change, which have been associated with increasing brain specialisation to language processing [7]. Dysregulation of deviance detection, as both suppression or enhancement of the event-related response, has been described as atypical neurological function [79]. More importantly, these early differences have been associated with downstream effects on social and non-social sound processing and language production [26, 49, 113]. Taken together, auditory habituation and deviance detection could provide important insights into basic perceptual processing in infants with NF1, and help us understand whether disrupted low-level auditory processing is predictive of ASD diagnosis in toddlerhood or appear as a cumulative risk factor across several neurodevelopmental conditions [29, 30, 46, 58].

In the current study, we examined age-related changes in auditory repetition suppression and change detection responses in infants with typical development (TD) and those diagnosed with NF1. We assessed infants at 5 and 10 months, which is a sensitive period for auditory development, as well as the comprehension and production of speech [95, 111]. Further, these processes occur prior to the onset of behavioural symptoms associated with neurodevelopmental conditions including ASD and ADHD, which holds great promise for early detection and implementation of effective interventions during the period of high brain plasticity. We presented infants with trains composed of three repeated vowels and then one deviant (either a change in the Vowel category or a change in its pitch), separated by an inter-train interval jittered between 3 and 5 seconds (simplified from a more complex design [23]). We indexed repetition suppression by comparing neural responses to the first and the second standard, and change detection by comparing the second standards with the deviants (reducing the influence of orienting effects from the first standard and preparatory effects from the third). As responses to the third standard could have additional influences from a recovery response [85], changes between the second and third repeated standard stimulus were examined as a secondary question in the SM. In some previous studies examining auditory processing and habituation the decrease in neural response between the first and second standards has been referred to as the fast decay response [93].

In the typically developing population, we expected a clear reduction in response between repetition of the first and second standard vowel sound at 5 and 10 months of age. We additionally expected a stronger change detection response (increased ERP negativity) towards pitch change at 5 months, and towards Vowel category change at 10 months, to reflect increasing specialisation to language processing [7]. Further, we predicted age-related changes in the localisation of repetition suppression and change detection responses between 5 and 10 months, to reflect increasing specialisation for sound processing towards the mature frontally driven response [88]. We predicted that infants with NF1 would show reduced or absent repetition suppression and change detection responses relative to typically developing infants at both 5 and 10 months of age. Finally, we examined whether individual differences in auditory change responses related to variation in three key phenotypes at a follow-up visit at 14 months: (a) language ability [107]; (b) ASD-relevant early behaviours as measured by the Autism Observation Scale for Infants [11]; and (c) early ADHD-relevant behaviours as measured by the Infant Behaviour Questionnaire-Revised [89], as a test of specificity [102];). We predicted there would be a positive association between a weaker repetition suppression and deviance detection response, reduced language ability and ASD-relevant behaviours.

## Methods

### Participants

We recruited 25 infants with NF1 and 52 typically developing (TD) infants into a longitudinal study running from 2013 to 2019. Some NF1 infants missed the 5-month entry point and entered at 10 months due to delays with families receiving the diagnosis (expert consultations take longer to be scheduled and attended). Infants were enrolled in the study if they either had Neurofibromatosis Type 1 or were typically developing. EEG was collected at 5 and 10 months, and a range of phenotypic measures were collected at a 14-month follow-up visit. We defined typical development (TD) as infants who had no first-degree relatives with a diagnosis of ASD or ADHD, with parents reporting no developmental concerns. These TD infants were recruited from a volunteer database at the Centre for Brain and Cognitive Development, Birkbeck, University of London. Inclusion criteria included full-term birth (gestational age greater than 36 weeks). At the time of enrolment, none of the infants in this cohort had a known medical or developmental condition. Participants in our NF1 cohort were recruited through local medical and genetic centres. All participants had their diagnosis confirmed via molecular testing of cord blood samples or clinical diagnosis based on NIH consensus criteria [105] and had no other developmental concerns at the time of the visits.

Informed written consent was provided by the parent(s) prior to the commencement of the study. The testing only took place if the infants were in a content and alert state. Ethical approval was granted by the National Research Ethics Service and the Research Ethics Committee of the Department of Psychological Sciences, Birkbeck, University of London. Participant families were reimbursed expenses for travel, subsistence and overnight stay if required. Infants were given a certificate and t-shirt after each visit.

**Fig. 1:**
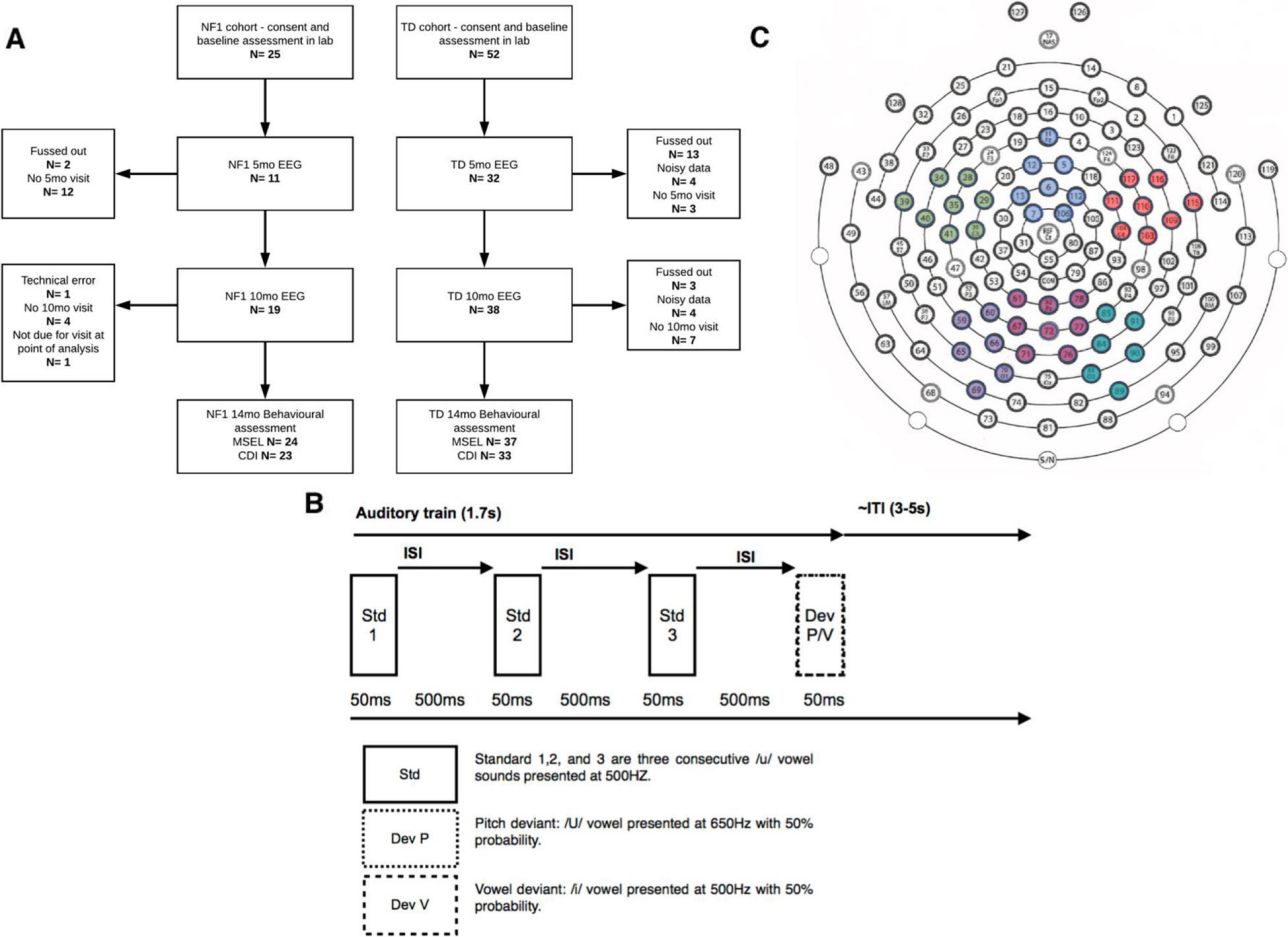
Auditory Task Methodology. (**a**) Participant numbers at each cohort, time point and task with attrition rates and reasons. Data was analysed using all infants with sufficient data at each time point; for information, the total number of infants who provided artefact-free EEG at both time points was NF1 *n* = 8; and TD *n* = 25. (**b)** ‘Train of vowels’ task design. Three (500 ms) auditory Standards were presented before a Deviant stimulus (500ms) followed by an ITI of ~ 2–3 s. (**c)** Electrodes chosen for analyses, with different colours depicting frontal left (dark green), frontal central (blue), frontal right (red), posterior left (purple), posterior central (maroon) and posterior right areas (turquoise)

A number of infants were excluded from the final EEG analyses for fussy behaviour (*n* = 15 and 3 at the 5- and 10-month time points respectively), noisy data (*n* = 4 and 4 at the 5- and 10-month time points respectively) or technical difficulties with the EEG protocol (*n* = 2 and 1 at the 5- and 10-month time points respectively) (see Fig. 1a and Table 1 for the final participant numbers and demographic breakdown by group at each time point).

**Table 1:** Participant characteristics; means (SD) of MSEL scores

	5 months		10 months	
	NF1	TD	NF1	TD
*n*	11	34	19	40
Sex	4 m, 7 f	15 m, 19 f	8 m, 11 f	17 m, 23 f
Age in days (SD)	191 (20)	167 (9)	308 (16)	317 (8)
Mullen composite score (SD)	67.09 (9.9)	89.73 (11.29)	78.47 (11.19)	90.18 (9.15)
Gross motor raw score	8 (.77)	8.45 (1.03)	10.95 (1.9)**	12.72 (1.47)
Visual reception raw score	6.64 (2.16)	7.33 (1.31)	11.89 (1.82)*	13.36 (1.4)
Fine motor raw score	5.82 (1.47)**	7 (1.7)	11.21 (2.23)**	14.51 (1.96)
Receptive language raw score	3.73 (1.56)*	5 (1.48)	8.53 (2.41)	8.74 (2.2)
Expressive language raw score	4.91 (.83)*	6.09 (.91)	8.84 (2.61)	8.56 (1.74)

*p < .05, **p < .001

### Stimuli

The auditory trains task consisted of sounds originally designed by [46]. Each train was composed of four consecutive 50-ms sounds with a 5-ms rise and fall time (Fig. 1b). The first three sounds in each train were repeated standards, and the fourth was a deviant. Standard sounds were all a /u/ vowel sound administered at 500 Hz. Deviants were either a Deviant Pitch (/U/ sound administered at 650 Hz) or Deviant Vowel sound (/i/ sound administered at 500 Hz) with 50% probability each. The inter-stimulus interval (ISI) was jittered at ~ 500 ms, and the inter-train interval was a random 3000–4000 ms. The sound intensity was 70dBA SPL. The sounds were presented for between 7 and 10 min, or until the infant became restless.

### Procedure

The auditory trains task was administered at the end of a battery of visual EEG tasks (not reported here). Infants were seated on their parents’/caregiver’s lap facing an experimenter. All testing took place in a sound-attenuated and electrically shielded room. Stimuli were presented via two speakers located behind a screen, placed 1 metre apart and approximately 1 metre away from the infant. Infants engaged in silent play (toys and/or bubbles) with a researcher. The task was still administered if infants fell asleep during any part of the EEG protocol (*n* = 2 for the NF1 group), as this does not affect the strength of the responses observed [21, 71].

### Fussy behaviour

Fussy behaviour is defined as excessive motion, behavioural signs of negative affect and avoidance behaviour that indicates the child is not enjoying participating in the experiment. Significant fussiness typically leads to poor quality or missing data. To maximise cross-researcher standardisation in responses to fussiness, we agreed a hierarchy of responses to maximise both data yield and participant comfort. Before the experiment, researchers asked parents to maximise their baby’s comfort by ensuring they were warm, fed, changed and seated comfortably. During the experiment, if a baby showed signs of fussiness (e.g. began to move more, show negative facial expressions, turn away from the stimuli) experimenters first addressed possible boredom by engaging the baby with a variety of silent toys (e.g. blowing bubbles). If fussiness continued, the parent was instructed to try (in this order) cuddling; holding hands; give baby something boring to hold (like a plastic teething ring); give baby a pacifier or snack; if all that did not work, a break was taken. If the parent wished to try again after the baby had calmed down, the experiment was resumed.

### Behavioural measures

#### Mullen Scales of Early Learning

The Mullen Scales of Early Learning (MSEL) [77] is a standardised measure that assesses developmental ability across five domains: Gross Motor, Visual Reception, Fine Motor, Receptive Language and Expressive Language. The MSEL was administered at all time points by trained researchers in the STAARS team. We used raw scores for subdomains to examine relations with neural activity;^1^ we present standard scores for overall developmental level for descriptive cohort comparisons.

#### Autism Observation Score for Infants

The Autism Observation Score for Infants (AOSI) [11] is a standardised research assessment that examines ASD traits in infants in the first two years of life. The measure involves a semi-structured play session between a researcher and the infant, examining such factors as social-communicative development, atypical sensorimotor behaviours and repetitive behaviours). Higher scores on this measure indicate greater ASD traits. Trained researchers administered the AOSI with our NF1 cohort at a follow-up 14-month time point.

#### Infant Behaviour Questionnaire-Revised

To measure ADHD-related early behaviours, we used the Activity Level subscale of the Infant Behaviour Questionnaire-Revised [89]. Previous work has shown this subscale to show a reasonable predictive relationship with later ADHD traits [102]. Parents completed the Infant Behaviour Questionnaire-Revised (IBQ-R) at a 14 month follow-up time point. The IBQ-R is a parent-report questionnaire consisting of 191 items and 14 scales that relate to different aspects of infant temperament. On a 7-point Likert scale, parents rated infant’s behaviours from “Never” to “Always”. Items are summed to produce a raw score for different subscales, any infants that had more than 20% missing data (including instances where parents reported “not applicable”) were not included in the final analyses for the subscale.

#### MacArthur Bates Communicative Development Inventory

Parents completed the MacArthur Bates Communicative Development Inventory (CDI) long form [31] at a follow-up 14-month time point. The CDI measures early language abilities by assessing vocabulary comprehension and expression, use of gestures and grammar. The questionnaire is comprised of approximately 800 items on a 1–3-point scale, with parents indicating whether their child can understand/understand and say certain words/complete certain gestures. Item scores can then be summed to produce a “Comprehension” score and an “Expression” score.

### Language scores

We computed composite scores across the MSEL and CDI measures for “Language Comprehension” and “Language Production” separately. We chose to form composite scores such that each encompassed both parent report and observational measures, giving a more comprehensive assessment of the two language domains. This also reduces the issue of multiple comparisons that are raised when correlating with different subtests that are designed to measure the same skill set. To compute the Language Comprehension composite score, the raw scores from the Receptive Language subscale of the MSEL and the sum of ‘Words Understood’ from the CDI were normalised (*z*-scored) based on subtracting the mean and then dividing by the standard deviation of the TD group. The resulting *z*-scores for the Receptive Language and CDI scales were then averaged together to produce the ‘Language Comprehension’ composite. The ‘Language Production’ composite score was calculated in the same way, but using the Expressive Language subscale of the MSEL and the sum of the total of ‘Words Understands and Says’ on the CDI. These scores were calculated from a follow-up visit at 14 months of age (see Table S1 for participant characteristics and individual subsets of scores).

### EEG processing

We recorded EEG data with an EGI (Philips Neuro, Oregon, USA) 128-electrode Hydrocel Sensor Net, with the vertex electrode (approximate position of Cz in 10–20 co-ordinate system) acting as a reference online and the data sampled at 500 Hz. We applied a 0.1–30-Hz bandpass filter offline. We segmented trials (representing the neural response to one sound within a train) 100 ms before and 800 ms after stimulus onset (Standard 1, Standard 2, Standard 3, Deviant Pitch and Deviant Vowel). We visually inspected EEG data for artefacts at a single-trial level. Segments with pronounced artefacts such as gross motor movement, eye blinks and/or more than 25 bad channels (before interpolation) were excluded from the analysis. Infants with fewer than 10 trials in any condition were excluded from any analyses involving that condition (see Table 2 for the final average number of presented and retained trials after artefact detection across age and groups). Within the remaining segments, channels marked as having a noisy signal were interpolated with a clean signal from neighbouring channels using spline interpolation. Following this, the data was baseline corrected (− 100 ms to 0 ms, relative to sound onset), re-referenced to the average of all electrodes and averaged across trials (see Fig. 1c for electrodes chosen for analyses). A proportion of the NF1 group were presented with a longer version of the paradigm. As such, we truncated our pre-processing and analyses to the first ~ 120 trials for Standards 1, 2 and 3 and the first ~ 65 trials for Deviant Pitch and Vowel.

**Table 2:** Mean (SD) and range of the number of presented trials. Mean % (SD) retained after artefact detection

	Standard 1	Standard 2	Standard 3	Deviant Pitch	Deviant Vowel
**Presented**					
**5 months**					
**NF1**	89 (26.97) 54–125	88.55 (27.1) 53–125	88.55 (27.1) 53–125	41.91 (15.61) 19–62	46.27 (12.86) 30–67
**TD**	81.97 (26.46) 38–150	81.62 (26.58) 38–150	81.5 (26.64) 38–150	40.09 (14.21) 19–76	41.15 (13.55) 19–76
**10 months**					
**NF1**	81.05 (21.89) 40–119	81.79 (23.27) 41–124	79.84 (21.77) 38–115	41.16 (11.08) 21–61	41.26 (12.94) 24–67
**TD**	76.7 (17.8) 53–127	76.33 (17.79) 53–127	76.03 (17.76) 52–126	37.08 (8.84) 23–61	38.73 (10.52) 23–65
**Retained %**					
**5 months**					
**NF1**	59.72 (21.82)	55.57 (22.52)	55.05 (19.48)	61.7 (19.41)	59.83 (19.73)
**TD**	43.27 (14.42)	42.57 (13.25)	43.09 (14.26)	49.01 (16.36)	48.47 (13.61)
**10 months**					
**NF1**	63.97 (.47)	63.92 (.42)	63.96 (.35)	63.92 (.92)	63.8 (.86)
**TD**	56.26 (14.48)	55.55 (13.72)	54.91 (13.54)	55.83 (14.62)	58.85 (14.35)

### Data analysis methods

#### Neural response analyses

To address our key questions, we examined (a) overall responses to the first standard as a measure of basic auditory processing; (b) changes in response between the first and second standard as a measure of repetition suppression; and (c) changes in response between the second standard and the Deviant Pitch/Vowel category as a measure of change detection; finally, we examined (d) the difference in neural responses to the pitch change and vowel category deviants as a measure of auditory discrimination. Within each analysis, we examined both differences in the mean amplitude of the ERP waveform across 50-ms time windows (Analysis Type 1: Mean amplitude ERP analysis) and the onset of significant differences in the ERP waveform (Analysis Type 2: Autocorrelation analyses).

#### Analysis 1: Mean amplitude ERP analysis

Within each key question, separate linear mixed models (LMMs) with the following dependent variables were run: “Standard 1”, “Standard 1-Standard 2”, “Standard 2-Deviant Pitch”, “Standard 2-Deviant Vowel” and “Deviant Pitch-Deviant Vowel” for both the frontal and posterior regions analysed separately (because of the opposite polarity of waveforms). In order to examine effects of further subsequent repetition, we also conducted analyses for “Standard 2-Standard 3” (see SM1).

All LMMs used the following fixed factors: Age (5 months, 10 months), Group (typical likelihood, NF1), site (left, central, right) and Time (100–150 ms, 150–200 ms, 200–250 ms, 250–300 ms, 300–350 ms, 350–400 ms, 400–450 ms, 450–500 ms). The repeated covariance type was set as “compound symmetry” and the maximum likelihood estimate was used for each model (see Table S3 for a summary of significant results).

#### Analysis 2: Autocorrelation analysis

Autocorrelation analyses allow us to examine the exact time at which neural responses to two different conditions begin to significantly differ from each other. Whereas more traditional ERP analyses focus on differences across a previously specified time window (typically as a result of top down hypotheses) or require selection of a peak (often less clearly apparent in infant data), autocorrelation analyses allow for more data-driven estimates of the onset of condition differences.

For this autocorrelation analysis, we compared ERP responses within the condition contrasts b) to e) outlined above using paired *t*-tests at each sample point (2ms intervals) in the first 500ms post-sound-onset. We corrected for the autocorrelation of consecutive sample points using a Monte Carlo simulation approach [47, 74]. This method estimated the average first-order autocorrelation (set at lag 1 and was .98 for all datasets analysed) present in the real difference waveforms of our experimental conditions. Following this, the method produced 1000 datasets of randomly generated waveforms. Each simulated difference waveform had a mean and unit variance of zero at each time point, but the same level of autocorrelation as seen on average in the observed data. Each simulated dataset also had the same number of participants and time samples as in the real data. We then applied two-tailed one-sample *t*-tests (vs. zero; alpha = .05, uncorrected) to the simulated waveforms at each time point, recording significant vs. non-significant outcomes. In each of the 1000 simulations, the longest sequence of consecutive significant *t*-test outcomes was computed. The 95th percentile of that simulated distribution of “longest sequence lengths” was then used to determine a significant difference waveform in the real data; specifically, we noted any sequences of significant *t*-tests that exceeded this 95th percentile in the real data. This method thus avoids the difficulties associated with multiple comparisons and preserves the type I error rate at .05 for each difference waveform analysed.

Due to the constraints of the statistical assumptions of *t*-tests, we were restricted to comparing each age group separately (as not all participants would have data for both the 5 and 10-month time points). We collapsed across site (left, central, right) and analysed separately by region (frontal, posterior) (see Table S4 for a summary of significant results). We also conducted control analyses where each Group had the same number of participants (see SM2), with relatively similar results as those presented below.

### Relationship with later development

In order to examine relationships between auditory processing and later development, we first needed to reduce the current number of variables (so as to streamline the number of correlations). To do this, we conducted a factor analysis on the primary variables of interest from the auditory processing task (Standard 1, Standard 1-Standard 2, Standard 2-Deviant Pitch and Standard 2-Deviant Vowel; mean amplitudes averaged over 100–500 ms and over left, central and right areas; frontal and posterior separate for each condition; both age points entered together). We used the principle components method, with a direct oblimin rotation given the relatedness of the variables. Two components were extracted (both eigenvalues > 1) that were consistent with responses to deviant stimuli, and the Initial Response/Change to the Standards. The deviant stimuli response factor was comprised of Standard 2-Deviant Pitch and Standard 2-Deviant Vowel (both frontal and posterior), factor loadings of -.74 to .84 and explaining 43% of the variance. The Initial Response/Change to the Standards factor was comprised of Standard 1 and Standard 1-Standard 2 (both frontal and posterior), with factor loadings of -.56 to .9 and explaining 24% of the variance; positive factor loadings represent frontal variables and negative factor loadings denote posterior variable. Of note, this same factor structure was obtained if the two age points were examined separately (see SM3). We then used the factor scores at the level of each individual in a number of correlation analyses that investigated relationships with language development and ASD and ADHD traits.

We measured infants’ language skills on two measures: the MSEL [77] and the MacArthur Bates Communicative Development Index (CDI) long form [31] and used our composite scores of Language Comprehension and Language Production in later analyses.

## Results

### Developmental ability

We conducted a MANCOVA on our MSEL scores (with age in days as a covariate; see Table 1), comparing raw scores between the two groups (asterisks indicate significant Group differences). This showed that the NF1 group showed lower language skills at 5 months, but not 10 months, than the TD group, and lower cognitive and motor skills at 10 months.

Table 2 shows the number of presented and retained trials for each Group at the 5- and 10-month time points. Generally, the NF1 group had a higher percentage of retained trials; as such, we conducted control analyses with retained trial numbers as a covariate.

#### Response to sudden sound (Standard 1)

##### Frontal

**Fig. 2:**
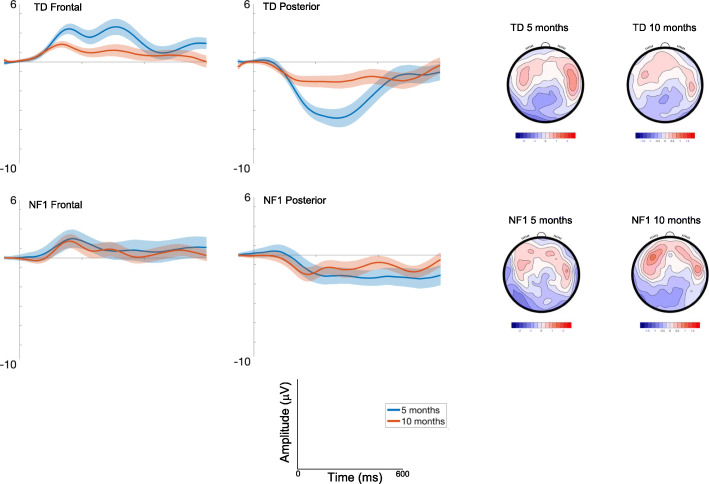
ERP waveforms for Standard 1; shading indicates the standard deviation. Topographic plots depict the absolute amplitude across the scalp in μV

Amplitudes decreased with Age [F(1, 2416) = 25.43, *p* < .001, *η*p^2^ = .01], fluctuated over the waveform (effect of Time: Fig. 2; [F(7, 2426) = 5.56, *p* <. 001, *η*p^2^ = .02]) and were smaller centrally than laterally [F(2, 2426) = 30.07, *p* < .001, *η*p^2^ = .02; Fig. 2]. There were no Group differences [F(1, 70) = .91, *p* = .34, *η*p^2^ = .01; see Table S3 for a summary of all analysis statistics].

##### Posterior

Responses decreased with Age [F(1, 2445) = 18.7, *p* < .001, *η*p^2^ = .008], were greater over the left area [F(2, 2426) = 3.29, *p* = .04, *η*p^2^ = .003]; and varied over the waveform [effect of Time; F(7, 2426).= 6.5, *p* < .001, *η*p^2^ = .02; Fig. 2]. The effect of Age varied by Group [F(1, 2445) = 4.51, *p* = .03, *η*p^2^ = .001; with number of trials retained covaried F = 6.8, *p* = .009, *η*p^2^ = .003], with pairwise comparisons indicating greater change in amplitude between 5 (M = − 3.53 μV, SE = .47 μV) and 10 months of age (M = − 1.88 μV, SE = .46 μV) in the TD group (mean diff = − 1.65μV, df = 2495, *p* < .001, CI = − 2.14 to − 1.17μV) than in the NF1 group (5 months: M = − 2.18 μV, SE = .72 μV, 10 months : M = − 1.61 μV, SE = .67 μV; mean diff = − .57 μV, df = 2415, *p* = .21, CI = − 1.45 to .32 μV); Bonferroni corrected to *p* = .013.

#### Effect of repetition (Standard 1**-**Standard 2)

##### Frontal

**Fig. 3:**
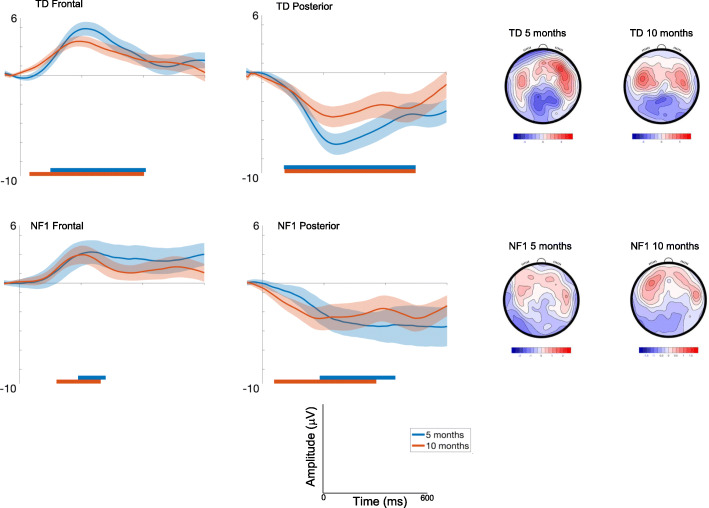
ERP difference waveforms for Standard 1-Standard 2; shading indicates the standard deviation. Topographic plots depict the difference in absolute amplitude between conditions across the scalp in μV. Solid horizontal bars indicate periods where the two conditions significantly differed from each other

Amplitudes slightly decreased with Age at trend level [F(1, 2305) = 3.36, *p* = .07, *η*p^2^ = .001], and varied across the waveform with the most pronounced difference between conditions around 200ms (effect of Time; [F(7, 2426) = 10.71, *p* < .001, *η*p^2^ = .03]) and were greatest over right areas [F(2, 2426) = 33.49, *p* < .001, *η*p^2^ = .03; Fig. 3]. There were no Group differences [F(1, 71) = .01, *p* = .92, *η*p^2^ = .01].

##### Posterior

Amplitudes were greater over central areas [F(2, 2427) = 6.97, *p* = .001, *η*p^2^ = .006] and changed over the waveform with the most pronounced difference between conditions around 200ms [effect of Time; F(7, 2427).= 2.17, *p* = .03, *η*p^2^ = .006]. Age effects varied by Group [F(1, 2455) = 5.86, *p* = .02, *η*p^2^ = .002; with number of trials retained covaried F = 10.48, *p* = .001, *η*p^2^ = .004], with decreasing effects of repetition on the neural response in the TD group between 5 (M = − 5.13 μV, SE = .71μV) and 10 months (M = − 3.77 μV, SE = .7μV) (mean diff = − 1.37μV, df = 2496, *p* < .001, CI = − 2.09 to − .64μV); responses in NF1 group did not change (5 months: M = − 3.39 μV, SE = 1.09; 10 months M = − 3.87 μV, SE = 1.02) (mean diff = .48μV, df = 2429, *p* = .67, CI = − .83 to 1.79μV); Bonferroni corrected to *p* = .013.

Autocorrelation results showed the onset and offset times of the periods of the waveform in which there were significant differences in the neural response between different conditions (see Table S3). For both diagnostic groups, 10-month-old infants showed differences earlier in the time course than 5-month-old infants (Fig. 3, Table S3). The duration of significant waveform differences was also longer in the 10-month-olds and in posterior than frontal regions more generally across both groups. When examining group differences, for the frontal region the TD group displayed earlier latencies of onset of significant responses to repetition than the NF1 group at both ages (Table S4). This pattern was also observed over posterior regions at 5 months. However, over posterior regions at 10 months, repetition detection was earlier in the NF1 group (by around 35 ms).

#### Detection of a change in pitch (Standard 2**-**Deviant Pitch)

##### Frontal

**Fig. 4:**
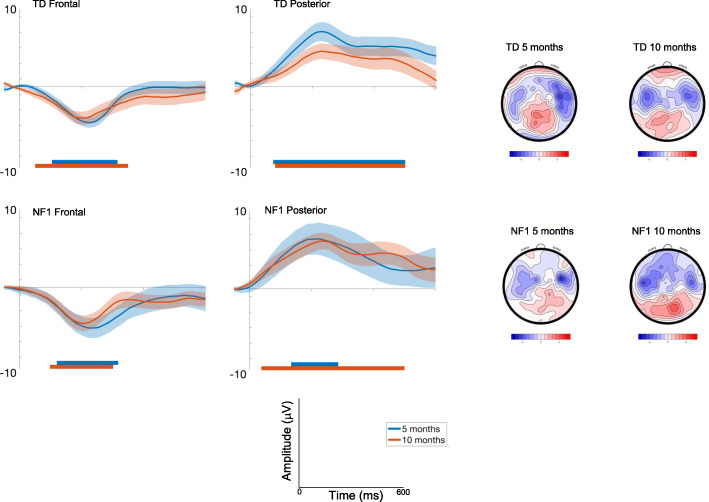
ERP difference waveforms for Standard 2-Deviant Pitch; shading indicates the standard deviation. Topographic plots depict the difference in absolute amplitude between conditions across the scalp in μV. Solid horizontal bars indicate periods where the two conditions significantly differed from each other

Amplitudes slightly decreased with Age at trend level [F(1, 2393) = 3.63, *p* = .06, *η*p^2^ = .002] and were greater over right areas [F(2, 2355) = 43.52, *p* < .001, *η*p^2^ = .04; see Table S2 and S3]. Responses varied across the waveform such that the most pronounced difference between conditions was around 200ms [effect of Time; Fig. 4; F(7, 2355) = 17.99, *p* < .001, *η*p^2^ = .05]. Responses did not vary by Group [F(1, 69) = .72, *p* = .4, *η*p^2^ = .01].

##### Posterior

Amplitudes slightly decreased with Age at the trend level [F(1, 2421) = 3.03, *p* = .08, *η*p^2^ = .001], were greater over central than lateral regions [F(2, 2353) = 8.29, *p* < .001, *η*p^2^ = .007]; and varied over the waveform such that differentiation between conditions was greater around 200ms [effect of Time; F(7, 2354).= 4.83, *p* < .001, *η*p^2^ = .01] (see Fig. 4). The effect of Age differed by Group [F(1, 2421) = 15.35, *p* < .001, *η*p^2^ = .006; with number of trials retained covaried F = 11.84, *p* = .001, *η*p^2^ = .005], with pairwise comparisons demonstrating decreasing effects of deviance on posterior waveforms between 5 (M = 5.29 μV, SE = .8) and 10 months (M = 3.2 μV, SE = .8μV) in the TD group (mean diff = 2.09μV, df = 2421, *p* < .001, CI = 1.37 to 2.81μV); responses in the NF1 group were largely stable (5 months: M = 4.19 μV, SE = 1.25; 10 months: M = 5 μV, SE = 1.18; mean diff = − .8μV, df = 2420, *p* = .21, CI = − 2.06 to .46μV); Bonferroni corrected to *p* = .013.

Once again, autocorrelation results showed that 10-month-old infants demonstrated differences earlier in the time course than 5-month-old infants (Fig. 4, Table S4) for pitch detection. The duration of significant waveform differences was also longer in the 10-month-olds and in posterior regions more generally across both groups. When examining group differences, for the frontal region the TD group displayed earlier latencies of onset of significant responses to pitch change than the NF1 group at both ages (Table S4). This pattern was also observed over posterior regions at 5 months. However, over posterior regions at 10 months, the detection of a pitch deviant was earlier in the NF1 group (by around 40 ms).

#### Detection of a change in vowel category (Standard 2**-**Deviant Vowel)

##### Frontal

**Fig. 5:**
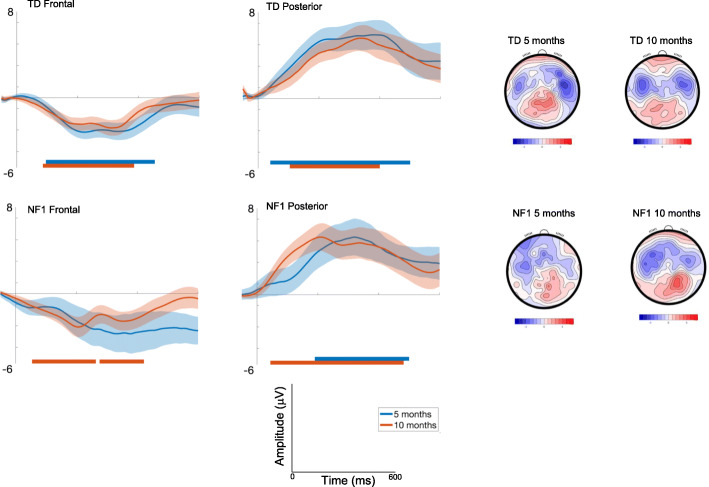
ERP difference waveforms for Standard 2-Deviant Vowel; shading indicates the standard deviation. Topographic plots depict the difference in absolute amplitude between conditions across the scalp in μV. Solid horizontal bars indicate periods where the two conditions significantly differed from each other

Responses were greater over the left areas [F(2, 2378) = 21.34, *p* < .001, *η*p^2^ = .02]; and varied over the waveform such that the difference between conditions was most pronounced around 300ms [effect of Time; F(7, 2378) = 7.59, *p* < .001, *η*p^2^ = .02]; Fig. 5. There were no significant Group differences [F(1, 70) = .93, *p* = .44, *η*p^2^ = .01].

##### Posterior

Responses were greatest over central areas [F(2, 2378) = 13.33, *p* < .001, *η*p^2^ = .01]; and varied over the waveform such that the difference between conditions was most pronounced around 200-300ms [effect of Time; F(7, 2378) = 3.66, *p* = .001, *η*p^2^ = .01]; Fig. 5. There were no significant Group differences [F(1, 70) = .26, *p* = .61, *η*p^2^ = .003].

Here, autocorrelation analyses demonstrated that 10-month-olds showed differences earlier in the time course than 5-month-old infants (Fig. 5, Table S4) for vowel category detection. The duration of significant waveform differences was also generally longer in the 10-month-olds and in posterior regions more generally across both groups. When examining group differences, at 5 months significant differences were seen in the TD group but not the NF1 group. At 10 months, the NF1 group also showed earlier differentiation over frontal and posterior regions at 10 months (NF1 earlier by around 30 and 60ms respectively).

#### Auditory discrimination (Deviant Pitch-Deviant Vowel category)

Given that we observe difference between our groups in their ability to detect deviant stimuli, we examined if there were direct differences in the processing of the Deviant Pitch-Deviant Vowel category.

##### Frontal

**Fig. 6:**
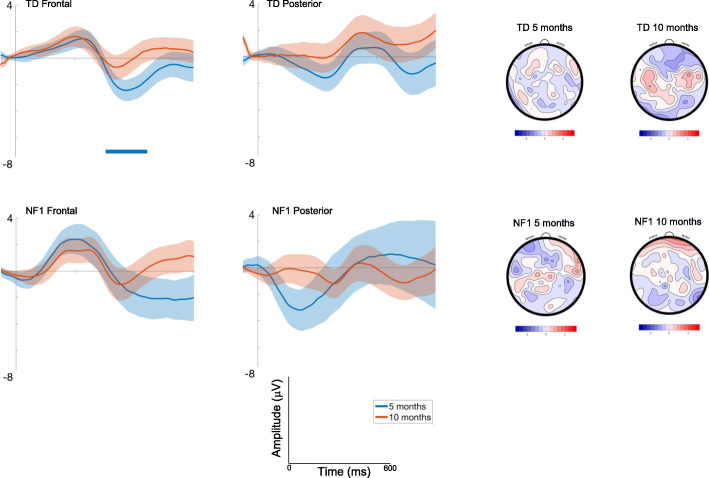
ERP difference waveforms for Deviant Pitch-Deviant Vowel; shading indicates the standard deviation. Topographic plots depict the difference in absolute amplitude between conditions across the scalp in μV. Solid horizontal bars indicate periods where the two conditions significantly differed from each other

Responses varied over the waveform such that the difference between conditions was most pronounced around 150ms and 300ms [effect of Time; F(7, 2404) = 9.87, *p* < .001, *η*p^2^ = .03]; Fig. 6, with the younger infants demonstrating a more negative deflection to the change in pitch than the change in vowel, an effect that was diminished at 10 months [effect of Age; F(1, 2407) = 12.09, *p* = .001, *η*p^2^ = .005].

##### Posterior

Responses were greatest over central areas [F(2, 2403) = 3.74, *p* = .02, *η*p^2^ = .003]; and varied over the waveform such that the difference was most pronounced around 100ms [effect of Time; F(7, 2403) = 5.9, *p* < .001, *η*p^2^ = .02]; Fig. 6. Finally, we also found a significant interaction of Age and Group [F(1, 2467) = 6.46, *p* = .01, ηp^2^ = .003; with number of trials retained covaried F = 12.07, *p* = .001, *η*p^2^ = .005], with pairwise comparisons demonstrating the response to pitch becoming more positive than to vowel between 5 (M = − .29 μV, SE = .78) and 10 months (M = .81 μV, SE = .77 μV) in the TD group (mean diff = − 1.1 μV, df = 2471, *p* = .003, CI = − 1.83 to − .37μV); responses in the NF1 group changed in the opposite direction (5 months: M = .52 μV, SE = 1.23; 10 months: M = − .34 μV, SE = 1.15; mean diff = .87μV, df = 2461, *p* = .2, CI = − .46 to 2.2 μV); Bonferroni corrected to *p* = .013.

In the autocorrelation analyses the only significant difference seen was in the TD group at 5 months, with a more negative response to the Deviant Vowel category than the Deviant Pitch stimulus (from 304 to 444 ms) (see Fig. 6). This effect remained in our matched participant analysis (see SM2; Table S5).

### Summary

In sum, LMM analyses indicate that there are age-related changes in posterior brain activity in the typically developing group that are not present in the NF1 group. These effects were present for responses to sudden-onset sounds, habituation of response to a repeated sound, and detection of a change in pitch but not a change in vowel category. Autocorrelation analyses indicate slower responses over the frontal region in the NF1 group, but often enhanced or more rapid responses over the posterior region. This was the case particularly at 10 months and in response to Deviant Vowel category stimuli in the NF1 group. Results were robust to controlling for trial numbers and repeating autocorrelation analyses using groups with matched samples (see SM 2; Table S5).

#### Relationship to later development

To consolidate our predictor variables, we examined relations between factor scores (Deviant Stimuli Response factor, Initial/Change to the Standards) and later development. Of note, we also conducted LMMs on our factor scores (Deviant Stimuli Response factor, Initial/Change to the Standards) with the following fixed factors: Age (5 months, 10 months) and Group (Typical likelihood, NF1). The repeated covariance type was set as ‘compound symmetry’ and the maximum likelihood estimate was used for the model. We found no significant effect of Age [Deviant Response: F(1, 64) = .74, *p* = .39; *η*_p_^2^ = .01; Initial/Change to Standards: F(1, 74) = 1.04, *p* = .31, *η*_p_^2^ = .01], no effect of Group [Deviant Response: F(1, 67) = .18, *p* = .67; *η*_p_^2^ = .003; Initial/Change to Standards: F(1, 61) = .77, *p* = .38, *η*_p_^2^ = .01] and no interaction of Group by Age [Deviant Response: F(1, 64) = .19, *p* = .67; *η*_p_^2^ = .003; Initial/Change to Standards: F(1, 74) = .06, *p* = .81, *η*_p_^2^ = .001].

We did not find any significant relationships between participants’ Deviant Stimuli Response and Initial Response/Change to the Standards scores and Language Comprehension [*r*(44) = − .07, *p* = .65 and *r*(44) = .008, *p* = .96 respectively] or Language Production composite scores [*r*(44) = − .03, *p* = .85 and *r*(44) = .05, *p* = .75 respectively]. Interestingly, when examining the language subscales individually, we found that increased Initial Response/Change to the Standards at 10 months was associated with better Receptive Language scores on the MSEL at 14 months; however, this should be treated with caution given the multiple comparisons [*r*(42) = .33, *p* = .03; see SM4).

**Fig. 7:**
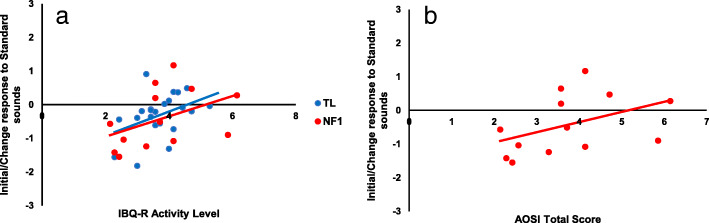
Scatter plots of Initial/Change response to Standard sounds at 10 months against IBQ-R Activity Level at 14 months in TL and NF1 cohorts (Panel **a**) and AOSI Total Scores in NF1 infants (Panel **a**)

Whilst we did not find significant associations between our neural Factor scores and our Language composites, we did find that a greater Initial Response/Change to Standards at 10 months was related to both greater levels of activity on the IBQ-R [*r*(36) = .43, *p* = .01] as well as more ASD traits as measured on the AOSI [*r*(12) = .61, *p* = .04] (see Fig. 7).

In order to scrutinise whether the neural response was more related to ASD or ADHD traits, we conducted two partial correlations. When controlling for IBQ-R Activity Level, the relationship between the Initial Response/Change to the Standards at 10 months and AOSI Raw Scores dropped to a trend level though still with a strong effect size [*r*(9) = .49, *p* = .12]. When controlling for AOSI Raw Score, the relationship between Initial/Change Response to Standards at 10 months and IBQ-R Activity Level became non-significant with a much reduced effect size, though it is important to note the small sample size [*r*(9) = .11, *p* = .74]. As such, the Initial Response/Change to Standards factor at 10 months seems to be more closely related to early ASD traits. Further analyses split by brain region and condition indicated that the associations with the AOSI were broadly present for both the initial response to the standards and repetition suppression to the second standard across both frontal and posterior areas of the brain (SM5).

## Discussion

This is the first study to examine the neural correlates of auditory processing in infants with NF1. Results showed that infants with NF1 are able to discriminate between different auditory stimuli (e.g. they are able to detect auditory repetition and detect auditory stimulus change). However, response profiles differed between the groups. Whilst effects over posterior regions decreased in magnitude between 5 and 10 months of age in typically developing infants (resulting in a more mature frontally centred response), this developmental change was less pronounced in the NF1 group. Further, typically developing infants largely differentiated stimulus repetition or change earlier in the waveform than the NF1 group over frontal (but not posterior regions), suggestive of processing delays in infants with NF1. Individual differences in these neural responses did not relate to Language Comprehension or Expressive Language, but did relate to early ASD and ADHD-related behaviours.

### Response to repetition

Both our NF1 and TD cohorts showed suppression of the neural response to Standard 2 relative to Standard 1. Given our paradigm, the effects of repetition likely reflect both sensory gating and fast-decay forms of habituation. Sensory gating refers to the suppression of a neural response to a rapidly repeated stimulus and has been observed as early as 50ms after auditory stimulus onset in infants aged between 1 and 4 months [51, 52, 57]. In adults, sensory gating is most common between 100 and 200 ms post-stimulus onset, consistent with the peak timing of our observed difference waves [10, 104]. Habituation is a broader process that includes decreased neural responses to repetition over short and longer timescales and likely contributes to the suppression of responses across the waveform in addition to subsequent responses to further stimulus repetition that are sometimes used to assess asymptotes in responding (e.g. the Standard 2-Standard 3 difference scores reported in the SM). Thus, the overall nature of the event-related response to auditory repetition was present in both groups, confirming our paradigm worked as expected.

The topography and timing of the effects of repetition were however different between infants with and without NF1. Specifically, over frontal regions, repetition suppression emerged later in the waveform for the NF1 than TD group at both ages. However, the pattern was different over posterior regions. Whilst at 5 months the TD group were again faster to detect repetition than the NF1 group over posterior regions, at 10 months, the NF1 group were faster. Further, the linear mixed models showed developmental decreases in the repetition effect over posterior regions between 5 and 10 months in the TD group but not the NF1 group. We believe these patterns of neural response to be demonstrative of less mature (or altered) repetition detection. Specifically, we posit that there is a shift in the topography of the neural response in typical development from a more global to a more localised fronto-central mature profile. This is evidenced by both the diminishing effect of suppression with age in the linear mixed model analyses over posterior regions in the TD group; and the suppression effect happening faster at 10 months than 5 months over frontal areas in the TD group, whilst the suppression effect doesn’t change in timing over posterior regions. This profile is altered in the NF1 group. This group does not show changes over posterior regions in the magnitude of the suppression effect and show a faster suppression effect across both frontal and posterior regions at 10 than 5 months. The pattern of these changes suggests that activity was becoming more frontally focused in the TD but not the NF1 group, reflecting a more mature topographic profile. Of note, this pattern of repetition suppression was specific to the change between the first and second standards; there were no group differences in responses to Standard 2 versus Standard 3 (SM1). This could indicate specificity of these differences to more rapid forms of suppression; however, it is important to note the limitation that the Standard 2/Standard 3 comparison may be affected by preparatory responses during the third standard (since it was always followed by a deviant).

The magnitude of the response to the first stimulus and the degree of suppression to the second stimulus were associated with later behavioural traits of ASD (measured with the AOSI). Although significant associations were initially seen with a PCA-formed composite of EEG repetition measures that did not itself vary by age or group, we also saw associations with responses to the first standard and the Standard 1-Standard 2 change over frontal and posterior scalp regions analysed separately. The AOSI predicts later ASD relatively well [12, 38], but not perfectly. This suggests that alterations in sensory processing may be related to the likelihood of infants with NF1 going on to a later ASD diagnosis, although further longitudinal follow-up with a larger sample would be required to test this hypothesis. Interestingly, previous work with 8-month-old infants with later ASD demonstrated differentiation of auditory stimuli over posterior areas only [60]. This differentiation response typically becomes more fronto-central with age [42], which is consistent with the current findings from our TD group. In comparison, our NF1 group show a more delayed or atypical pattern of neural response across age. In general, habituation as a mechanism has been shown to be altered in ASD [50, 72], with atypical patterns seen in infants as young as 3 months who are at an elevated likelihood of developing the disorder [25]. Another possibility is that these effects relate to the heightened and prolonged startle response to even weak auditory stimuli sometimes observed in ASD [15, 59, 106]. Sensory processing is of particular interest in ASD given that 90% of individuals with the neurodevelopmental disorder report atypicalities [65], that are evident across a number of sensory systems [90]. Given that sensory systems undergo rapid development (both pre- and post-natally), it may be that early alterations in sensory processing may have a cascading effect on later developmental trajectories. As such, our results may reflect alterations in auditory sensory processing that predict the emergence of later ASD traits.

One note of caution is however that our results are not necessarily specific to later ASD symptoms. In the present dataset, we also observed an association between responses to the first and second stimuli and later ADHD-related traits (IBQ activity levels, which we have previously associated with later ADHD symptoms [102]. It is important to note that this association was also seen for both groups and thus may not relate to subsequent clinically significant ADHD traits, given that we would not expect elevated levels of ADHD within our TD cohort. The effect size of the relation between early auditory processing and later ASD traits remained stronger when covarying for activity level than in the reverse analysis, suggesting that there may be a closer mechanistic link to later ASD symptoms. However, we did not observe relations to two specifically hypothesised elements of the ASD phenotype that we thought might provide a developmental link between auditory processing and later symptoms (language skills and auditory sensory sensitivities). In addition to this, we must point out that the AOSI was not measured in our TD group, so it is difficult to determine if the same positive association would have been observed between the neural response to the first and second auditory stimulus and ASD traits in this cohort. However, research has indicated that infants with a typical likelihood of ASD score much lower on this behavioural assessment than those with an elevated likelihood of ASD (e.g. [40]), with the measure able to differentiate these cohorts from 12 months of age [114]. Taking these factors into consideration, further work will be required to evaluate whether early auditory processing differences are more generally linked to later neurodevelopmental difficulties or more specifically linked to particular phenotypes later on.

### Change detection

Alterations were also observed in the responses to a change in deviant category (vowel or pitch). Both changes produced characteristic fronto-central negative deflections, as well as positive-going deflections over posterior regions. For the change in pitch, the TD group showed a stable differentiation over fronto-central regions but a diminishing differentiation over the posterior cortex between 5 and 10 months. Further, the differentiation occurred earlier in the waveform over frontal regions at 10 than 5 months, but did not change over posterior regions. Again, this is potentially reflective of the emergence of the mature fronto-centrally focused MMN component in the TD infants. Previous research with adults has shown that the auditory MMN response is predominantly observed over the auditory cortex with some recruitment of the frontal and parietal regions ([1, 39, 41, 73]. Indeed, a frontocentral topographic presentation has been observed in young children [41, 42] and infants, although it is typically broader and more central (for a review, see Cheour et al. [17]). Thus, the increasingly fronto-central topography observed in the TD group may be an expected shift towards a more mature pattern.

Similar to the effects of repetition, the profile was different in the NF1 group. At 5 months, the NF1 group differentiated the change in pitch later in the waveform than the TD group over both posterior and frontal regions, suggestive of globally slower processing. Between 5 and 10 months, response latencies got faster in the NF1 group over posterior regions such that at 10 months of age the NF1 group were faster in differentiating stimuli that deviated in pitch than the TD group. Again, these effects suggest an alteration in the topographic specialisation of auditory processing in NF1. However, unlike responses to repetition, these effects did not relate to later measures of ASD traits. It is possible that these effects may be related to the enhanced pitch perception that has been shown in ASD [26], but we may need longer-term follow-up data to fully evaluate this possibility.

In terms of the differentiation of the vowel change stimulus, a significant period of differentiation was not observed over frontal regions until 10 months in the NF1 group but was present at 5 months in the TD group, which could be consistent with a developmental delay in NF1. However, we did not find this effect in a similar analysis performed on groups of a matched sample size. Thus, more research is required to determine the robustness of this finding in NF1. Further, the linear mixed models did not indicate significant group differences in response profiles. It may be that changes in responses to vowel categories would be observed later in development, where processing of this change has become more specialised. When directly contrasting changes in pitch and vowel categories, our autocorrelation analyses demonstrated that the TD group showed a significant window late in the waveform at 5 months of age in the frontal region where responses were more negative to the pitch than the vowel change; this effect had diminished by 10 months and was not present in the NF1 group. This may reflect age-related changes as infants shift to more mature processing where both types of deviants (pitch and vowel change) are processed in terms of probability rather than surface features. Interestingly, this was only seen in our TD group, which suggests alterations in the way the NF1 group detect changes in deviant categories. Consistent with this hypothesis, in the LMM, analyses examining the posterior region indicated that whilst the response to pitch was greater than to vowel by 10 months in the TD group, progression moved in the opposite direction for the NF1 group. Again, this may be consistent with a reduction in sensitivity to vowel change over posterior cortex with age in typical infants, with a movement to the more neurotypical fronto-central distribution,

A further avenue of research would be to investigate auditory processing in younger infants. Our present results are consistent with the NF1 group showing less mature auditory processing. As such, future research could study auditory processing in the first 2 years of life to map the protracted developmental trajectory of repetition and change detection. Additionally, the paradigm is easily adaptable to be used with other sensory modalities (e.g. the tactile domain) in order to determine if atypicalities in sensory processing are domain general or are specific to within a sensory system. Indeed, this may be a particularly pertinent area of investigation given the sensory sensitivities related to touch within the ASD literature [13, 33].

When examining the relationship between auditory processing and later development, we did not find a correlation between the neural response and either composite scores for Language Production or Language Comprehension. A possible explanation for this lack of relationship could be due to the age at which we were measuring language skills. It may be that 14 months of age is perhaps too young to get an accurate representation of language development, especially in terms of expressive language, and it may be more fruitful to examine this relationship later in development (e.g. toddlerhood). Other studies have found associations between neural responses to vowel or pitch change and language development [7, 101]. Alternatively, it may be that the more predictable paradigm used in the current study does not tap into language development in the same way as traditional MMN paradigms. This could then potentially speak to underlying mechanisms regarding temporal predictability in language development. In turn, it may be that traditional MMN paradigms are measuring unpredictability rather than detecting changes in auditory stimuli per se. Alternatively, it may be that by forming language composite scores (to reduce the issue of multiple comparisons) we are missing key elements of variability in our dataset. Indeed, when we decompose our Language Composites scores we see a positive association between the Initial/Change Response to Standard stimuli and Receptive Language on the MSEL. However, if corrected for multiple comparisons this would no longer be significant and thus may be treated with caution.

Further research in this area could be translational in nature. Auditory paradigms, in general, lend themselves to being used translationally with a wide range of species (both asleep and awake) due to the passive nature of the task. Non-human animal models have been particularly informative in NF1 research [22, 29, 30, 43, 103], demonstrating impairments in attention, habituation, motor co-ordination and visual-spatial learning. As the sensory systems are tightly coupled, and research has well established the modulatory effect of attention on sensory processing [78, 112], this may be a fruitful avenue of further research when investigating NF1. Given the ease of use of the current paradigm, and that no NF1 mouse models of auditory processing currently exist, this task is primed for use with a number of different species as well as in the youngest human primates.

Further to the use of the current paradigm with animal participants, if replicated the task could also potentially be used in a battery of screening measures for infants at elevated likelihood of developing neurodevelopmental disorders such as ASD or ADHD. With the relative ease and accessibility of EEG as a methodology, and the particular ease of the task with infant populations, it would be feasible to use the measure with infants even in the neonatal period. Additionally, the nature of the task also means that it is possible to administer with older populations, which would allow for more frequent evaluations and detailed longitudinal comparisons. Whilst the current task used very short, simple phonemes that infants may not have recognised as language components, it would be possible to incorporate more complex language stimuli to tease apart low level auditory processing and language processing. Finally, to further test the hypothesised shift from posteriorly to frontally focused neural activity, future avenues of research may be inclined to co-register fMRI and EEG for source analyses. This may allow greater mechanistic insights into the neural systems that underpin the scalp-level changes apparent in the present study.

## Conclusions

We present the first evidence of atypical neural correlates of auditory processing in infants with Neurofibromatosis Type 1. We found that infants with NF1 showed slower neural detection of repetition or change, and diminished developmental change in the scalp profile of neural responses. Whilst auditory responses were not related to later language development, an increased neural response to an initial auditory stimulus was related to more ASD-relevant behaviours at 14 months. Taken together, this auditory neural response may indicate alterations in early sensory processing and specialisation that could provide valuable options for developmental screening within groups of infants with NF1.

## Supplementary Information

**Additional file 1.**

## Data Availability

At present, the datasets generated and/or analysed during the current study are not publicly available due to confidentiality constraints within our ethical approvals. These datasets can be accessed via The BASIS Network (http://www.basisnetwork.org/) upon completion of the requisite data access and sharing protocols.

## Abbreviations

ASD: Autism spectrum disorder
ADHD: Attention deficit hyperactivity disorder
AOSI: Autism Observation Scale in Infants
CDI: Communicative Development Inventory
EDEN: early development in Neurofibromatosis Type 1
EEG: Electroencephalography
ERP: Event-related potential
IBQ-R: Infant Behaviour Questionnaire-Revised
LMM: Linear mixed model
MSEL: Mullen Scales of Early Learning
NF1: Neurofibromatosis type 1
STAARS: Studying Autism and ADHD Risks
TD: Typically developing

## Footnotes

1 To allow for the greatest level of replicability and consistency across examiners, our guidelines for Mullen scoring include only behaviours that are captured on camera (so can be confirmed by a second/third researcher if necessary) within the Mullen session. For example, if an infant demonstrates babbling throughout the rest of the testing day (i.e. during another task or a lunch break), but not during the specific Mullen administration session, we would not score this infant as being able to produce babbling sounds on the Expressive Language scale. To further ensure the fidelity of the scoring, a second fully trained researcher watches the administration in real time (via a video feed) and consensus discussions take place after the testing session. These strict administration and scoring guidelines make the US norms less applicable. As such, we decided to use the raw scores of the four subdomains (as opposed to the *T*-scores, presented in Table 1).
