# Supplementary material for: Early differences in auditory processing relate to Autism Spectrum Disorder traits in infants with Neurofibromatosis Type I
Source: J Neurodev Disord. 2021 May 28;13:22. doi: 10.1186/s11689-021-09364-3 (PMC8161667; doi:10.1186/s11689-021-09364-3)
Supplement: Supplementary file 1 — Additional file 1. [file 11689_2021_9364_MOESM1_ESM.docx]

TITLE: AUDITORY PROCESSING IN INFANTS WITH NEUROFIBROMATOSIS TYPE I: Supplementary Materials

Jannath Begum Ali^1*^, Anna Kolesnik-Taylor^1,2*^, Isabel Quiroz^1^, Luke Mason^1^, Shruti Garg^3^, Jonathan Green^2^, Mark H. Johnson^1,4^ & Emily J. H. Jones^1^ and the STAARS and EDEN Teams^φ^

*Joint first authors

1. Analysis 1 Results: **Effect of repetition (Standard 2-Standard 3)**

We also examined the habituation response between the second and third Standard stimuli (both frontally and posteriorly) via an LMM. We use the following fixed factors: Age (5 months, 10 months), Group (Typical likelihood, NF1), Site (Left, Central, Right) and Time (100-150ms, 150-200ms, 200-250ms, 250-300ms, 300-350ms, 350-400ms, 400-450ms, 450-500ms). The repeated covariance type was set as ‘compound symmetry’ and the maximum likelihood estimate was used for the model.

Frontal: Amplitudes decreased with age [F(1, 2424) = 11.68, p = .001, *η_p_*^2^ = .005], varied across the waveform [F(7, 2427) = 4.57, p <. 001, *η_p_*^2^ = .01; see Figure S1] and were greatest over the left hemisphere [F(2, 2427) = 5.8, p < .001, *η_p_*^2^ = .005]. There were no group differences [F(1, 71) = .005, p = .94, *η_p_*^2^ = 0].

Posterior: Amplitudes were greater over central areas [F(2, 2427) = 5.33, p = .005, *η_p_*^2^ = .002]. Age effects varied by group [F(1, 2425) = 4.1, p = .04, *η_p_*^2^ = .002; with number of trials retained covaried F = 9.34, p = .002, *η_p_*^2^ = .004], with greater differentiation in the neural response in the Nf1 group (5 months: M = .91μV, SE = .97; 10 months M = 2.18μV, SE = .9) (mean diff = -1.27µV, df = 2387, p = .04, CI = -2.5µV to -.04µV; though this did not survive Bonferroni correction, p = .013) than in the TD group between 5 (M = 2.95μV, SE = .63µV) and 10 months (M = 2.76μV, SE = .9µV) (mean diff = .19µV, df = 2493, p = .6, CI = -.4µV to .87µV).


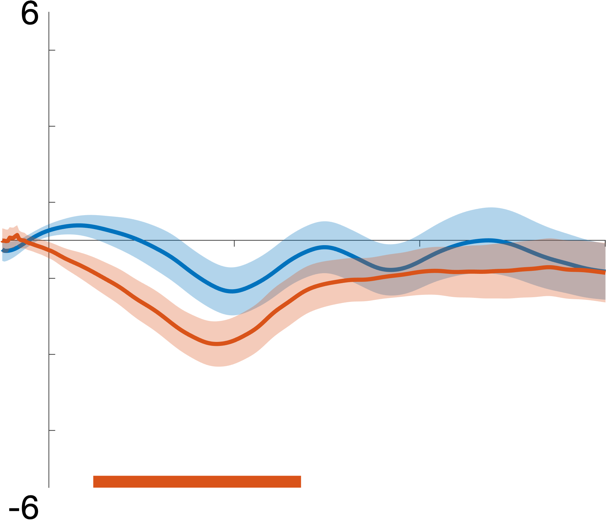

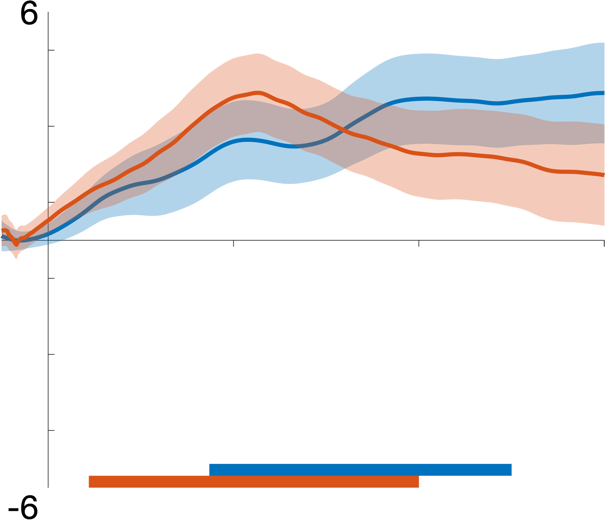


TD Frontal

TD Posterior


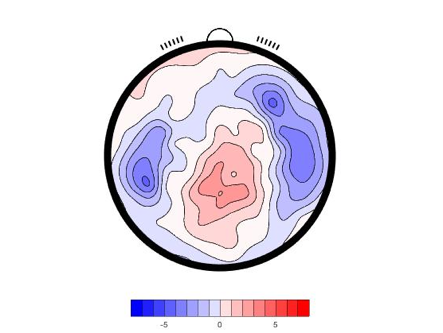


TD 5 months


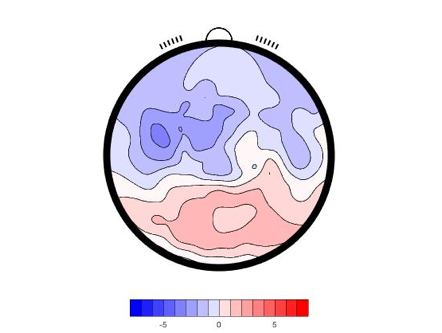


TD 10 months


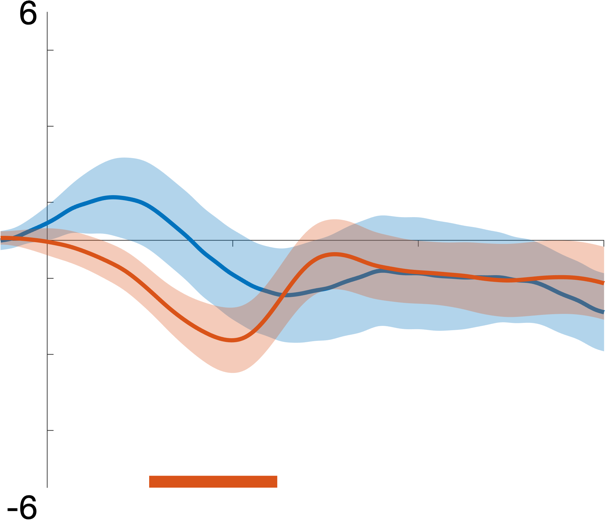

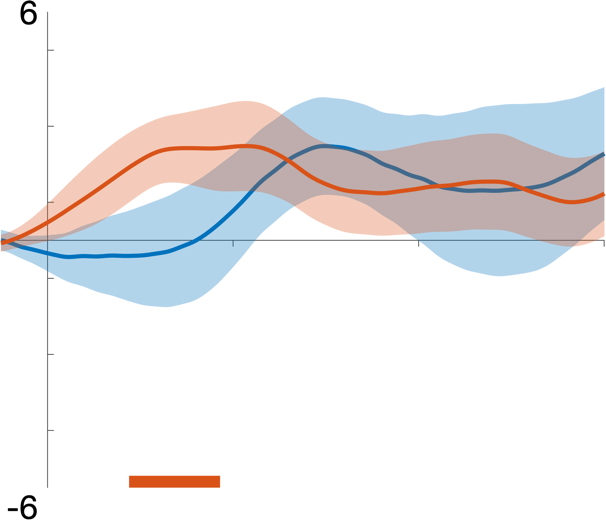

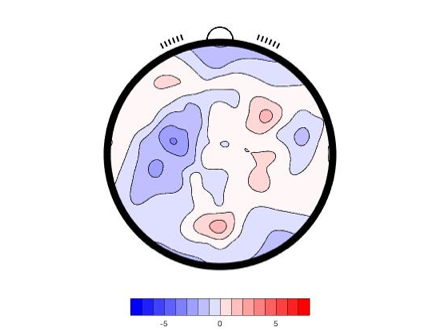


NF1 5 months


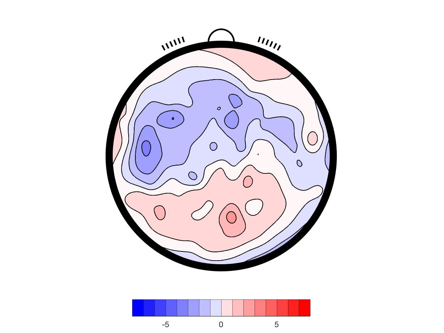


NF1 10 months

NF1 Frontal

NF1 Posterior

Amplitude (μV)

Time (ms)


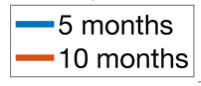


600

0

Figure S1: Figure showing ERP difference waveforms for Standard 2-Standard 3, shading indicates the standard deviation. Topographic plots depict the difference in absolute amplitude between conditions across the scalp in μV. Solid horizontal bars indicate periods where the two conditions significantly differed from each other.

1. Analysis 2: Autocorrelation analyses with matched samples

It could be argued that one potential explanation for the earlier differentiation between conditions we see in the TD group may be due to the larger sample size in this group. It may be that the variability is reduced in this group and there is a smaller standard error compared to the NF1 group; the NF1 group may be reaching statistical significance at later time windows because of this increased variability. In order to fully explore this potential explanation, we conducted a further autocorrelation analysis with equal numbers of participants in both the TD and NF1 groups to see if our results remained the same. We matched participants based on sex and age in days. The number of infants in our NF1 group did not change from the original analyses (n = 11 and 19 at 5- and 10-months respectively), however the TD group was reduced to these numbers.

We ran the same comparisons as in the original analyses and largely found the same effects with this matched sample (see Table S6 below). Broadly, the TD group still demonstrated faster differentiation of auditory stimuli, albeit this was more comparable to the NF1 group at 5 months. Further, the TD group remained slower at differentiating deviant stimuli over frontal (vowel deviant) and posterior (pitch and vowel deviants) regions. Interestingly, the TD group at 5 months were no longer detecting repetition (Standard 2 vs Standard 3) or the vowel deviant, which is more comparable with the NF1 group at the same age. Given these effects seemed to be driven by the increased sample size of our TD group, we do not overstate these results in the broader discussion of our findings.

1. Analysis 3: PCA of neural responses split by Age

Our original PCA in the main text examined neural response values collapsed across Group and Age. To address whether there may have been differences in these factor scores due to Age, we conducted separate factor analyses for the 5 and 10-month-olds respectively. We used the same parameters and variables as in the main text. Our primary variables of interest were: Standard 1, Standard 1-Standard 2, Standard 2-Deviant Pitch and Standard 2-Deviant Vowel; mean amplitudes averaged over 100-500ms and over Left, Central and Right areas; Frontal and Posterior separate for each condition. We used the principle components method, with a direct oblimin rotation given the relatedness of the variables. We derived 2 factors (both eigenvalues > 1) that corresponded to ‘Initial/Change Response to Standards’ and ‘Deviant Stimuli Response’ separately for both age groups.

For the 5 month time point, the ‘Deviant Stimuli Response’ factor was comprised of Standard 2-Deviant Pitch and Standard 2-Deviant Vowel (both frontal and posterior), with factor loadings of -.94 to .74 and explaining 40.66% of the variance. The Initial Response/Change to the Standards factor was comprised of Standard 1 and Standard 1-Standard 2 (both frontal and posterior), with factor loadings of -.89 to .91 and explaining 27.04% of the variance. Negative factor loadings corresponded to Posterior variables, whereas positive factor loadings corresponded to Frontal variables.

For the 10 month time points, the ‘Deviant Stimuli Response’ factor was comprised of Standard 2-Deviant Pitch and Standard 2-Deviant Vowel (both frontal and posterior), with factor loadings of -.89 to .81 and explaining 50.41% of the variance. The Initial Response/Change to the Standards factor was comprised of Standard 1 and Standard 1-Standard 2 (both frontal and posterior), with factor loadings of -.91 to .88 and explaining 23.21% of the variance. These results are extremely similar to those reported in the main manuscript when the PCA was conducted on the whole cohort (across age group). Further, the fact that the variables load on the same factors would suggest a common, underlying source. As such, we feel justified in conducting the PCA on all participants across age group.

1. Analysis 4: Relationship with raw Language scores

In order to further examine whether there were any relationships between our neural factor scores (Deviant Stimuli Response and Initial/Change Response to Standards) and language development, we also conducted correlations between the factor scores at 10-months and the raw language scores that comprised our Language Comprehension and Production composites at 14-months. As such, we looked at the correlation between each Factor Score and the MSEL Expressive and Receptive Language Scales and the CDI ‘Understand’ and ‘Understand and Says’ totals.

We found that those with an increased Initial/Change Response to Standards score also demonstrated better Receptive Language scores on the MSEL [r(42) = .33, p = .03]. This was not the case for the CDI ‘Understands’ score [r(37) = -.08, p =.63], nor any of the Language Production raw scores [MSEL Expressive Language: r(42) = .18, p = .25; CDI ‘Understand and Says’: r(37) = .22, p = .2]. We found no relationship between the Deviant Stimuli Response factor and Language Comprehension [MSEL Receptive Language: r(42) = -.14, p = .37; CDI ‘Understands’: r(37) = .06, p = .71] or Language Production [MSEL Expressive Language: r(42) = .0, p = 1; CDI ‘Understand and Says’: r(37) = -.13, p = .46]. 5. Analysis 5: Relationship with ASD traits

To examine which of the variables contributed to the positive, significant relationship between the Initial/Change Response to Standards factor and the AOSI Total Score and IBQ Activity Level scores, we correlated the mean ‘Standard 1’ and ‘Standard 1 – Standard 2’ ERP values with the AOSI and IBQ variables. As the factor score combined both of these variables, we chose to use the separate mean ERP amplitude for each of these variables. Further, we also kept the ‘frontal’ and ‘posterior’ variables separate in this analysis, as issues of polarity reversals may not lend themselves to clarifying the underlying relationships between neural activity and later ASD traits. Examining these bivariate correlations (see Table S7 below), it seems that both the response to Standard 1 and the Change in response to Standard 2 contribute to the relationship between neural activity and later development (specifically the AOSI Total score and IBQ Activity level score).

| Table S7: Correlations between neural response at 10 months and measures of later development at 14 months | | | | |
| --- | --- | --- | --- | --- |
|  | Frontal Standard 1 | Frontal Standard 1 – Standard 2 | Posterior Standard 1 | Posterior Standard 1 – Standard 2 |
| AOSI Total Raw Score | r(12) = .7, p = .01 | r(12) = .61, p = .04 | r(12) = -.55, p = .07 | r(12) = -.58, p = .05 |
| IBQ-R Activity level | r(40) = .38, p = .02 | r(40) = .28, p = .08 | r(40) = -.32, p = .05 | r(40) = -.24, p = .14 |

1. SM Tables and Figures referred to in the main text
   1. Table S1

| **Table S1: Participant characteristics; means (SD) of raw Mullen and CDI scores by domain** | | |
| --- | --- | --- |
|  | 14 months | |
|  | NF1 | TD |
| n | 24 | 43 |
| Sex | 14f, 10m | 20f, 23m |
| Age in days (SD) | 449.17 (23.61) | 446.84 (15.47) |
| MSEL Receptive language Raw Score | 10.54 (2.89) | 13.54 (2.5) |
| MSEL Expressive language Raw Score | 11.58 (3.4) | 13.65 (3.83) |
| CDI ‘Understand’ | 87.96 (95.47) | 125.24 (85.86) |
| CDI ‘Understand and says’ | 7.96 (10.52) | 27.09 (36.72) |

- 1. Table S2

| **Table S2: Mean amplitude µV (SE) across Conditions and Laterality from 100-500ms** | | | |
| --- | --- | --- | --- |
|  | Left | Central | Right |
| Frontal |  |  |  |
| Habituation response |  |  |  |
| Standard 1 | 1.82 (.39) | .5 (.41) | 1.81 (.4) |
| Standard 2 | -1.1 (.35) | -.64 (.41) | -1.32 (.35) |
| Standard 3 | .37 (.27) | .07 (.33) | -.4 (.34) |
| Change detection |  |  |  |
| Deviant Pitch | 1.76 (.36) | .32 (.48) | 1.76 (.47) |
| Deviant Vowel | 1.85 (.38) | .75 (.45) | 1.08 (.44) |
| Posterior |  |  |  |
| Habituation response |  |  |  |
| Standard 1 | -2.74 (.46) | -2.4 (.48) | -2.19 (.47) |
| Standard 2 | 1.88 (.52) | 2.53 (.53) | 1.56 (.47) |
| Standard 3 | -.78 (.37) | -.47 (.38) | -.45 (.36) |
| Change detection |  |  |  |
| Deviant Pitch | -2.78 (.61) | -2.69 (.55) | -2.46 (.46) |
| Deviant Vowel | -2.67 (.71) | -3.13 (.62) | -2.41 (.52) |

6.3 Table S3:

| **Table S3: Summary statistics (at significant or trend level) of LMM analysis in main text** | | | | | | | |
| --- | --- | --- | --- | --- | --- | --- | --- |
| **Standard 1** | | | | | | | |
| **Frontal** | | | | **Posterior** | | | |
| **Effect** | **df** | **F** | **p** | **Effect** | **df** | **F** | **p** |
| Age | 1, 2416 | 25.43 | <.001 | Age | 1, 2445 | 18.7 | .<001 |
| Laterality | 2, 2426 | 30.07 | <.001 | Laterality | 2, 2426 | 3.29 | .04 |
| Time | 7, 2426 | 5.56 | <.001 | Time | 7, 2426 | 6.5 | <.001 |
|  |  |  |  | Age*Group | 1, 2445 | 4.51 | .03 |
| **Standard 1 vs Standard 2** | | | | | | | |
| **Frontal** | | | | **Posterior** | | | |
| **Effect** | **df** | **F** | **p** | **Effect** | **df** | **F** | **p** |
| Age | 1, 2305 | 3.36 | .07 | Laterality | 2, 2427 | 6.97 | .001 |
| Laterality | 2, 2426 | 33.49 | <.001 | Time | 7, 2427 | 2.17 | .03 |
| Time | 7, 2426 | 10.71 | <.001 | Age*Group | 1, 2455 | 5.86 | .02 |
| **Standard 2 vs Deviant Pitch** | | | | | | | |
| **Frontal** | | | | **Posterior** | | | |
| **Effect** | **df** | **F** | **p** | **Effect** | **df** | **F** | **p** |
| Age | 1, 2393 | 3.63 | .06 | Age | 1, 2421 | 3.03 | .08 |
| Laterality | 2, 2355 | 43.52 | <.001 | Laterality | 2, 2353 | 8.29 | <.001 |
| Time | 7, 2355 | 17.99 | <.001 | Time | 7, 2354 | 4.83 | <.001 |
|  |  |  |  | Age*Group | 1, 2421 | 15.35 | <.001 |
| **Standard 2 vs Deviant Vowel** | | | | | | | |
| **Frontal** | | | | **Posterior** | | | |
| **Effect** | **df** | **F** | **p** | **Effect** | **df** | **F** | **p** |
| Laterality | 2, 2378 | 21.34 | <.001 | Laterality | 2, 2378 | 13.33 | <.001 |
| Time | 7, 2378 | 7.59 | <.001 | Time | 7, 2378 | 3.66 | .001 |
|  |  |  |  |  |  |  |  |
| **Deviant Pitch vs Deviant Vowel** | | | | | | | |
| **Frontal** | | | | **Posterior** | | | |
| **Effect** | **df** | **F** | **p** | **Effect** | **df** | **F** | **p** |
| Time | 7, 2404 | 9.87 | <.001 | Time | 7, 2403 | 5.9 | <.001 |
| Age | 1, 2407 | 12.09 | .001 | Laterality | 2, 2403 | 3.41 | .03 |
|  |  |  |  | Age*Group | 1, 2467 | 6.46 | .01 |

6.4: Table S4

| **Table S4: Summary of statistics from Autocorrelation analysis in main text** | | | | | |
| --- | --- | --- | --- | --- | --- |
| **Standard 1 vs Standard 2** | | | | | |
|  | | 5 months | | 10 months | |
| Group(s) | Region | Critical sequence length (ms) | Period of significance (ms) | Critical sequence length (ms) | Period of significance (ms) |
| TD | Frontal | 52 | 98-408 | 37 | 30-402 |
| NF1 | Frontal | 43 | 190-280 | 45 | 120-264 |
| TD | Posterior | 47 | 72-500 | 44 | 74-500 |
| NF1 | Posterior | 41 | 186-432 | 30 | 38-370 |
| TD vs NF1 | Frontal | - | - | - | - |
| TD vs NF1 | Posterior | - | - | - | - |
| **Standard 2 vs Standard 3** | | | | | |
|  | | 5 months | | 10 months | |
| Group(s) | Region | Critical sequence length (ms) | Period of significance (ms) | Critical sequence length (ms) | Period of significance (ms) |
| TD | Frontal | - | - | 49 | 48-272 |
| NF1 | Frontal | - | - | 45 | 110-248 |
| TD | Posterior | 42 | 174-500 | 40 | 44-400 |
| NF1 | Posterior | - | - | 34 | 88-186 |
| TD vs NF1 | Frontal | - | - | - | - |
| TD vs NF1 | Posterior | - | - | - | - |
| **Standard 2 vs Deviant Pitch** | | | | | |
|  | | 5 months | | 10 months | |
| Group(s) | Region | Critical sequence length (ms) | Period of significance (ms) | Critical sequence length (ms) | Period of significance (ms) |
| TD | Frontal | 56 | 104-316 | 50 | 50-350 |
| NF1 | Frontal | 45 | 120-318 | 45 | 98-302 |
| TD | Posterior | 43 | 74-500 | 45 | 80-500 |
| NF1 | Posterior | 37 | 134-286 | 32 | 38-500 |
| TD vs NF1 | Frontal | - | - | - | - |
| TD vs NF1 | Posterior | - | - | - | - |
| **Standard 2 vs Deviant Vowel** | | | | | |
|  | | 5 months | | 10 months | |
| Group(s) | Region | Critical sequence length (ms) | Period of significance (ms) | Critical sequence length (ms) | Period of significance (ms) |
| TD | Frontal | 41 | 96-454 | 49 | 86-386 |
| NF1 | Frontal | - | - | 34 | 54-264, 276-422 |
| TD | Posterior | 39 | 40-500 | 48 | 104-400 |
| NF1 | Posterior | 43 | 190-500 | 31 | 44-482 |
| TD vs NF1 | Frontal | - | - | - | - |
| TD vs NF1 | Posterior | - | - | - | - |
| **Deviant Pitch vs Deviant Vowel** | | | | | |
|  |  | 5 months | | 10 months | |
| Group(s) | Region | Critical sequence length (ms) | Period of significance (ms) | Critical sequence length (ms) | Period of significance (ms) |
| TD | Frontal | 58 | 304-444 | - | - |
| NF1 | Frontal | - | - | - | - |
| TD | Posterior | - | - | - | - |
| NF1 | Posterior | - | - | - | - |
| TD vs NF1 | Frontal | - | - | - | - |
| TD vs NF1 | Posterior | - | - | - | - |

6.5 Table S5

| **Table S5: Summary of statistics from Autocorrelation analysis – matched participants (SM Analysis 2)** | | | | | |
| --- | --- | --- | --- | --- | --- |
| **Standard 1 vs Standard 2** | | | | | |
|  | | 5 months | | 10 months | |
| Group(s) | Region | Critical sequence length (ms) | Period of significance (ms) | Critical sequence length (ms) | Period of significance (ms) |
| TD | Frontal | 43 | 142-278 | 40 | 0-312 |
| NF1 | Frontal | 43 | 190-280 | 45 | 120-264 |
| TD | Posterior | 55 | 150-288 | 32 | 0-500 |
| NF1 | Posterior | 41 | 186-432 | 30 | 38-370 |
| TD vs NF1 | Frontal | - | - | - | - |
| TD vs NF1 | Posterior | - | - | - | - |
| **Standard 2 vs Standard 3** | | | | | |
|  | | 5 months | | 10 months | |
| Group(s) | Region | Critical sequence length (ms) | Period of significance (ms) | Critical sequence length (ms) | Period of significance (ms) |
| TD | Frontal | - | - | 41 | 52-248 |
| NF1 | Frontal | - | - | 45 | 110-248 |
| TD | Posterior | - | - | 31 | 50-500 |
| NF1 | Posterior | - | - | 34 | 88-186 |
| TD vs NF1 | Frontal | - | - | - | - |
| TD vs NF1 | Posterior | - | - | - | - |
| **Standard 2 vs Deviant Pitch** | | | | | |
|  | | 5 months | | 10 months | |
| Group(s) | Region | Critical sequence length (ms) | Period of significance (ms) | Critical sequence length (ms) | Period of significance (ms) |
| TD | Frontal | 47 | 124-282 | 47 | 50-302 |
| NF1 | Frontal | 45 | 120-318 | 45 | 98-302 |
| TD | Posterior | 40 | 118-500 | 32 | 48-464 |
| NF1 | Posterior | 37 | 134-286 | 32 | 38-500 |
| TD vs NF1 | Frontal | - | - | - | - |
| TD vs NF1 | Posterior | - | - | - | - |
| **Standard 2 vs Deviant Vowel** | | | | | |
|  | | 5 months | | 10 months | |
| Group(s) | Region | Critical sequence length (ms) | Period of significance (ms) | Critical sequence length (ms) | Period of significance (ms) |
| TD | Frontal |  |  | 37 | 64-248 |
| NF1 | Frontal | - | - | 34 | 54-264, 276-422 |
| TD | Posterior | 40 | 186-450 | 36 | 122-478 |
| NF1 | Posterior | 43 | 190-500 | 31 | 44-482 |
| TD vs NF1 | Frontal | - | - | - | - |
| TD vs NF1 | Posterior | - | - | - | - |
| **Deviant Pitch vs Deviant Vowel** | | | | | |
|  |  | 5 months | | 10 months | |
| Group(s) | Region | Critical sequence length (ms) | Period of significance (ms) | Critical sequence length (ms) | Period of significance (ms) |
| TD | Frontal | 51 | 116-236 | - | - |
| NF1 | Frontal | - | - | - | - |
| TD | Posterior | - | - | - | - |
| NF1 | Posterior | - | - | - | - |
| TD vs NF1 | Frontal | - | - | - | - |
| TD vs NF1 | Posterior | - | - | - | - |

Correspondence concerning the article should be addressed to Jannath Begum Ali; Centre for Brain and Cognitive Development, Henry Wellcome Building, Birkbeck, University of London, Malet Street, WC1E 7HX. [Jannath.begum@bbk.ac.uk](mailto:Jannath.begum@bbk.ac.uk), 02076316931
